# *Zfat*-Deficiency Results in a Loss of CD3ζ Phosphorylation with Dysregulation of ERK and Egr Activities Leading to Impaired Positive Selection

**DOI:** 10.1371/journal.pone.0076254

**Published:** 2013-10-03

**Authors:** Masahiro Ogawa, Tadashi Okamura, Shuhei Ishikura, Keiko Doi, Hiroshi Matsuzaki, Yoko Tanaka, Takeharu Ota, Kunihiro Hayakawa, Harumi Suzuki, Toshiyuki Tsunoda, Takehiko Sasazuki, Senji Shirasawa

**Affiliations:** 1 Department of Cell Biology, Faculty of Medicine, Fukuoka University, Jonan-ku, Fukuoka, Japan; 2 Central Research Institute for Advanced Molecular Medicine, Fukuoka University, Jonan-ku, Fukuoka, Japan; 3 Division of Animal Models, Department of Infectious Diseases, Research Institute, National Center for Global Health and Medicine, Tokyo, Japan; 4 Department of Immunology and Pathology, National Institute for Global Health and Medicine, Chiba, Japan; 5 Institute for Advanced Study, Kyushu University, Fukuoka, Japan; University of Alberta, Canada

## Abstract

The human *ZFAT* gene was originally identified as a susceptibility gene for autoimmune thyroid disease. Mouse Zfat is a critical transcriptional regulator for primitive hematopoiesis and required for peripheral T cell homeostasis. However, its physiological roles in T cell development remain poorly understood. Here, we generated *Zfat* ^f/f^-*LckCre* mice and demonstrated that T cell-specific *Zfat*-deletion in *Zfat* ^f/f^-*LckCre* mice resulted in a reduction in the number of CD4^+^CD8^+^double-positive (DP) cells, CD4^+^single positive cells and CD8^+^single positive cells. Indeed, in *Zfat* ^f/f^-*LckCre* DP cells, positive selection was severely impaired. Defects of positive selection in *Zfat*-deficient thymocytes were not restored in the presence of the exogenous TCR by using TCR-transgenic mice. Furthermore, *Zfat*-deficient DP cells showed a loss of CD3ζ phosphorylation in response to T cell antigen receptor (TCR)-stimulation concomitant with dysregulation of extracellular signal-related kinase (ERK) and early growth response protein (Egr) activities. These results demonstrate that Zfat is required for proper regulation of the TCR-proximal signalings, and is a crucial molecule for positive selection through ERK and Egr activities, thus suggesting that a full understanding of the precise molecular mechanisms of Zfat will provide deeper insight into T cell development and immune regulation.

## Introduction

*ZFAT* was originally identified as a candidate susceptibility gene for autoimmune thyroid disease [1]. *ZFAT* contains 18 zinc-finger domains and one AT-hook and is an evolutionally conserved gene from fish to humans, and the Zfat protein is highly expressed in the thymus and the spleen in adult mice [2]. We previously reported that *Zfat*-deficient (*Zfat* ^−/−^) mice are embryonic lethal by embryonic day 8.5, and Zfat is a critical transcriptional regulator for primitive hematopoiesis [3], and that ZFAT is functionally involved in the regulation of apoptosis of mouse embryonic fibroblasts and MOLT−4 cells [4], [5].

Just recently, we generated *Zfat* ^f/f^ *-Cd4Cre* mice [6], and showed that *Zfat*-deficient mice exhibited a reduction in the number of peripheral T cells with decreased surface expression of IL-7Rα and T cell antigen receptor (TCR)-stimulation-induced expression of CD25 and IL-2, indicating that Zfat is required for peripheral T cell homeostasis [6]. On the other hand, genetic variants of *ZFAT* have also been reported to be associated with adult height in Japanese and Korean population [7], [8] and several common diseases including hypertension and cancer [9], [10]. Of great interest is that a genetic variant of *ZFAT* is reported to be strongly associated with interferon-β responsiveness in multiple sclerosis [11] and the severity of Hashimoto’s disease [12]. However, the exact functions of ZFAT during T cell development remain unknown.

T cells must be reactive to foreign pathogens, but tolerant to self-antigens. These features are formed during T cell development in the thymus [13]. CD4^+^CD8^+^double-positive (DP) cells expressing complete αβTCR complexes undergo positive selection, for differentiation into mature CD4^+^single positive (CD4SP) cells or CD8^+^single positive (CD8SP) cells [14]–[16]. DP cells that recognize self-peptide and major histocompatibility complex (pMHC) molecules with low affinities receive survival signals and differentiate into mature single positive cells; this process is known as positive selection. Accumulating evidence suggests that mitogen-activated protein kinase (MAPK) signaling pathways and the molecules in this pathway play critical roles in the regulation of the cellular fate during T cell development [17]. Extracellular signal-related kinase (ERK) is activated by phosphorylation through sequential activation of Ras, Raf and MEK transduced by TCR-stimulation, and proper ERK activation is essential for positive selection [18]–[20].

Egr1, Egr2 and Egr3 are zinc-finger transcription factors of the early growth response protein (Egr) family, and they are rapidly induced in response to TCR-stimulation [21]–[23]. *Egr1*- or *Egr2*-deficiency was reported to result in defects of positive selection and survival of T cells [24], [25]. *Egr3*-deficiency was reported to cause the impaired proliferation of thymocytes during transition from DN to DP cells [26]. Thus, TCR-stimulation-induced Egr expression is thought to be essential for the proper progression of T cell development in the thymus.

In this study, we generated *Zfat* ^f/f^-*LckCre* mice and showed that they exhibited a loss of CD3ζ phosphorylation with dysregulation of ERK and Egr activities leading to impaired positive selection. This is the first report demonstrating that Zfat is required for proper regulation of the TCR-proximal signalings, and is a crucial molecule for positive selection in the thymus.

## Results

### Reduction in the Number of Thymic DP, CD4SP and CD8SP cells in *Zfat* ^f/f^-*LckCre* Mice

**Figure 1:**
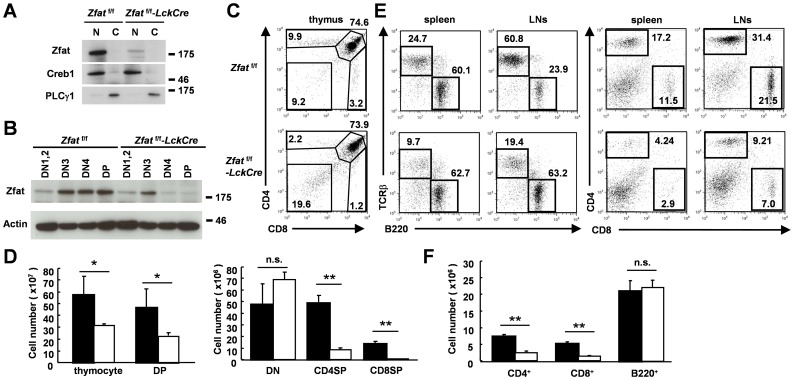
Reduction in the number of thymocytes and peripheral T cells in *Zfat* ^f/f^-*LckCre* mice. (A) An immunoblot of Zfat in nuclear (N) or cytoplasmic (C) fractions of thymocytes from *Zfat* ^f/f^ and *Zfat* ^f/f^-*LckCre* mice. CREB1 and PLCγ1 were used as controls of nuclear and cytoplasmic fractions, respectively. (B) An immunoblot of Zfat in DN1 and DN2 (DN1,2: CD4^−^CD8^−^CD44^+^), DN3 (CD4^−^CD8^−^CD25^+^CD44^−^), DN4 (CD4^−^CD8^−^CD25^−^CD44^−^) or DP thymocytes from *Zfat* ^f/f^ and *Zfat* ^f/f^-*LckCre* mice. Actin was used as a loading control. (C) Flow cytometry analysis of the surface expression of CD4 and CD8 on thymocytes from *Zfat* ^f/f^ (top) and *Zfat* ^f/f^-*LckCre* (bottom) mice at 6 to 7 weeks of age. Data are representative of three independent experiments. (D) Total numbers of thymocytes, thymic DP, DN, CD4SP or CD8SP cells from *Zfat* ^f/f^ (black bars) and *Zfat* ^f/f^-*LckCre* (white bars) mice at 6 to 7 weeks of age. The data are the mean ± standard deviation (s.d.); *n* = 6; * *P*<0.05; ** *P*<0.01; n.s., not significant. (E) Flow cytometry analysis of TCRβ^+^T cells and B220^+^B cells (left) or CD4^+^and CD8^+^T cells (right) in the spleen and LNs of *Zfat* ^f/f^ (top) and *Zfat* ^f/f^-*LckCre* (bottom) mice. Data are representative of three independent experiments. (F) Total numbers of CD4^+^T, CD8^+^T or B220^+^B cells in the spleen from *Zfat* ^f/f^ (black bars) and *Zfat* ^f/f^-*LckCre* (white bars) mice. The data are the mean ± s.d.; *n* = 6; ** *P*<0.01; n.s., not significant.

To clarify the physiological roles of Zfat in T cell development in the thymus, we crossed *Zfat* ^f/f^ mice [6] with *LckCre* transgenic mice. The deletion of Zfat in *Zfat* ^f/f^-*LckCre* thymocytes was confirmed by an immunoblot analysis. While Zfat was detected specifically in the nuclear fraction of *Zfat* ^f/f^ thymocytes, the protein was hardly observed in *Zfat* ^f/f^-*LckCre* thymocytes (Figure 1A), indicating the efficient deletion of Zfat in the thymocytes of *Zfat* ^f/f^-*LckCre* mice. During the transition of DN stages in the *Zfat* ^f/f^ mice, the Zfat expression levels in the DN1 (CD25^−^CD44^+^) and DN2 (CD25^+^CD44^+^) subsets was low, whereas Zfat expression in the DN3 (CD25^+^CD44^−^), DN4 (CD25^−^CD44^−^) and DP subsets was detected at a higher level (Figure 1B). In contrast, in *Zfat* ^f/f^-*LckCre* mice, Zfat expression in the DN3 subset was slightly decreased compared with that of *Zfat* ^f/f^ mice, whereas Zfat expression in DN4 and DP subsets was apparently decreased compared with that of *Zfat* ^f/f^ mice (Figure 1B). These results indicated that the Zfat expression in *Zfat* ^f/f^-*LckCre* mice was abolished at the DN4 stage.

In *Zfat* ^f/f^-*LckCre* mice, the proportions of CD4SP and CD8SP cells, but not DP cells, were remarkably reduced and the total number of thymocytes, DP cells, CD4SP cells and CD8SP cells was significantly decreased compared with that of *Zfat* ^f/f^ mice (Figure 1C, 1D). On the other hand, the proportion and total number of DN cells in *Zfat* ^f/f^-*LckCre* mice seemed to be slightly increased compared with those of *Zfat* ^f/f^ mice, however, the difference of the total number of DN cells was not statistically significant (Figure 1C, 1D). Consistent with the decreased proportions and total number of CD4SP and CD8SP cells in the *Zfat* ^f/f^-*LckCre* thymus, a reduction in the proportion of TCRβ^+^T cells in both the spleen and lymph nodes (LNs) was observed in *Zfat* ^f/f^-*LckCre* mice (Figure 1E). The proportion and the total number of CD4^+^or CD8^+^T cells in the spleen and LNs were significantly reduced in *Zfat* ^f/f^-*LckCre* mice in comparison to those of *Zfat* ^f/f^ mice (Figure 1E, 1F). These results demonstrated that *Zfat*-deficiency results in impaired T cell development in the thymus.

In the thymus, a slight reduction in the expression levels of IL-7Rα on CD4SP and CD8SP cells in *Zfat* ^f/f^-*LckCre* mice was observed compared with those from *Zfat* ^f/f^ mice (Figure S1A). However, a difference in the expression levels of Bcl-2, a pro-survival factor induced by IL-7-mediated signaling [27], [28], was not observed between the genotypes (Figure S1B), suggesting that the reduced expression of IL-7Rα did not play a functionally significant role in the reduction of T cells in the thymus of *Zfat* ^f/f^-*LckCre* mice.

### *Zfat-*deficiency did not Affect the Transition of thymic DN to DP Cells

**Figure 2:**
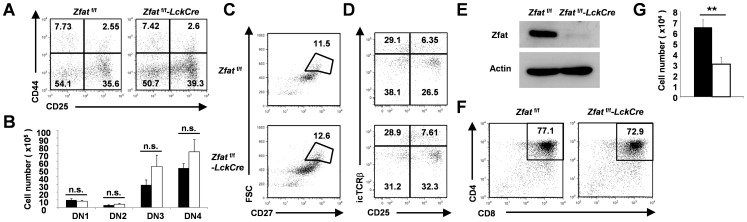
*Zfat*-deficiency did not affect the transition of thymic DN3 to DP cells. (A) Flow cytometry analysis of the surface expression of CD44 and CD25 on CD4^−^CD8^−^ DN thymocytes from *Zfat* ^f/f^ and *Zfat* ^f/f^-*LckCre* mice at 6 to 7 weeks of age. The numbers in the quadrants indicate the percent DN1 (upper left), DN2 (upper right), DN3 (lower right) or DN4 (lower left) subsets in DN thymocytes from the indicated genotypes. Data are representative of three independent experiments. (B) Total numbers of DN1, DN2, DN3 or DN4 cells from *Zfat* ^f/f^ (black bars, *n* = 3) and *Zfat* ^f/f^-*LckCre* (white bars, *n* = 3) mice at 6 to 7 weeks of age. The data are the mean ± standard deviation (s.d.); n.s., not significant. (C) Flow cytometry analysis of the surface expression of CD27 and forward scatter (FSC) of DN3 thymocytes. The gated area indicates DN3b (CD27^hi^FSC^hi^) subsets. Data are representative of three independent experiments. (D) Flow cytometry analysis of TCRβ-negative DN (CD4^−^CD8^−^TCRβ^−^) cells. The intracellular expression of TCRβ (icTCRβ) and surface expression of CD25 are shown. Data are representative of three independent experiments. (E-G) Sorted DN4 cells were co-cultured with Tst-4/DLL1 cells for 3 days and analyzed by an immunoblot of Zfat (E) and flow cytometry for the surface expression of CD4 and CD8 (F). The proportions (F) or total numbers (G) of CD4^+^CD8^+^DP thymocytes from *Zfat* ^f/f^ (top, black bar, *n* = 3) or *Zfat* ^f/f^-*LckCre* (bottom, white bar, *n* = 3) mice are shown. The data are the mean ± standard deviation (s.d.); ** P<0.01. * P<0.05.

As the proportion and number of DN cells in *Zfat* ^f/f^-*LckCre* mice seemed to be slightly increased compared with those of *Zfat* ^f/f^ mice (Figure 1C, 1D), we next analyzed whether *LckCre*-mediated *Zfat*-deficiency would affect the T cell development at the DN stage. The proportions and total numbers of the DN subsets, i.e., DN1, DN2, DN3 and DN4, in *Zfat* ^f/f^-*LckCre* mice did not show significant differences with those of *Zfat* ^f/f^ mice, although the total numbers of DN3 and DN4 subsets seemed to be slightly elevated in *Zfat* ^f/f^-*LckCre* mice (Figure 2A, 2B). At the DN3 stage, thymocytes undergo β-selection through the pre-TCR signaling, leading to the transition from DN3a (CD25^+^CD44^−^CD27^lo^FSC^lo^) to DN3b (CD25^+^CD44^−^CD27^hi^FSC^hi^) cells [29], [30]. The proportion of DN3b cells was comparable between the genotypes (Figure 2C), and the expression levels of intracellular TCRβ (icTCRβ) in *Zfat* ^f/f^-*LckCre* mice were comparable to those of *Zfat* ^f/f^ mice during the transition from the DN3 (CD25^+^icTCRβ^+^) to DN4 (CD25^−^icTCRβ^+^) stage (Figure 2D), which together indicated that the thymocytes in *Zfat* ^f/f^-*LckCre* mice normally passed through β-selection and transition from DN3 to DN4 cells.

We then addressed the transition from the DN4 to DP stage by analyzing an *ex vivo* culture system using Tst-4/DLL1 cells, which can support T cell development to the DP stage [31]. DN4 cells were sorted and then cultured on a monolayer of Tst-4/DLL1 cells. After 3 days of the culture, we confirmed a decline in Zfat expression in the thymocytes sorted from *Zfat* ^f/f^-*LckCre* thymus (Figure 2E), and observed the comparable proportions of the production of DP cells between *Zfat* ^f/f^ and *Zfat* ^f/f^-*LckCre* thymocytes (Figure 2F), indicating no blockade of the transition of DN4 to DP cells in *Zfat* ^f/f^-*LckCre* mice. Taken together, *Zfat-*deficiency did not affect the transition of thymic DN to DP cells including β-selection and transitions from DN3 to DN4 cells, and from DN4 to DP cells. In agreement with a decrease in DP cells *in vivo* (Figure 1D), however, the total number of the produced DP cells of *Zfat* ^f/f^-*LckCre* mice in *ex vivo* culture system was significantly reduced compared with that of *Zfat* ^f/f^ mice (Figure 2G), suggesting that there may be a defect in the proliferation or survival during the transition from DN to DP stage in the *Zfat*-deficient thymocytes.

### Impaired Positive Selection in Thymocytes of *Zfat* ^f/f^-*LckCre* Mice

**Figure 3:**
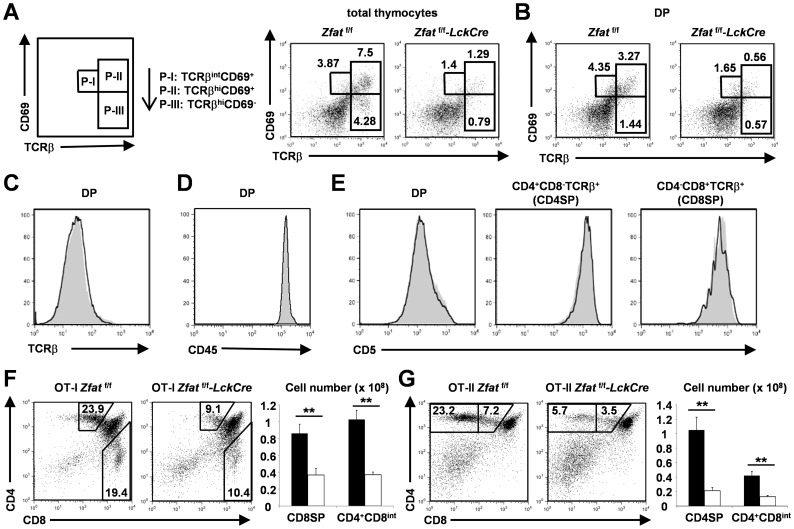
Impaired positive selection in *Zfat* ^f/f^-*LckCre* thymus. (A, B) A scheme of the intermediates of positive selection defined by changes in expression of TCRβ and CD69: TCRβ^int^CD69^+^(P-I), TCRβ^hi^CD69^+^(P-II) and TCRβ^hi^CD69^−^ (P-III) are generated sequentially as shown by arrows (A, left). Flow cytometry analysis of the surface expression of CD69 and TCRβ on total thymocytes (A) or DP cells (B) from the indicated genotypes at 6 to 7 weeks of age. The numbers indicate the proportion of the gated area. Data are representative of three independent experiments. (C-E) Surface expression of TCRβ on DP cells (C), CD45 on DP cells (D) or CD5 on DP, CD4^+^CD8^−^TCRβ^+^or CD4^−^CD8^+^TCRβ^+^cells (E) from *Zfat* ^f/f^ (gray-filled) and *Zfat* ^f/f^-*LckCre* (black line) mice at 6 to 7 weeks of age. Data are representative of three independent experiments. (F, G) Flow cytometry analysis for the surface expression of CD4 and CD8 on the thymocytes and total numbers of CD4^+^CD8^int^, CD8SP or CD4SP cells from OT-I *Zfat* ^f/f^ (black bar) and OT-I *Zfat* ^f/f^-*LckCre* mice (white bar) (F) or from OT-II *Zfat* ^f/f^ (black bar) and OT-II *Zfat* ^f/f^-*LckCre* mice (white bar) (G). The numbers indicate the proportion of the gated area. The data are the mean ± standard deviation (s.d.); *n* = 3; ** P<0.01. Data are representative of three independent experiments.

Loss of Zfat did not affect the DN to DP transition, in spite of significant reduction in the number of DP cells. We next analyzed an involvement of Zfat in positive selection of the DP cells. During the positive selection, TCRβ^int^CD69^−^ DP cells initially show a TCRβ^int^CD69^+^transitional phenotype (P-I; Figure 3A) after the TCR/pMHC interaction. After the positive selection, P-I cells become TCRβ^hi^CD69^+^cells (P-II; Figure 3A) and then differentiate into CD4SP or CD8SP cells (TCRβ^hi^CD69^−^) (P-III; Figure 3A) [20]. A considerable reduction in the proportion of P-I cells in thymocytes (1.4% versus 3.87%, respectively, Figure 3A) and DP cells (1.65% versus 4.35%, respectively, Figure 3B) from *Zfat* ^f/f^-*LckCre* mice was observed compared with that of *Zfat* ^f/f^ mice, indicating the existence of impaired positive selection in the *Zfat*-deficient DP thymocytes. However, no obvious alterations in the rearrangements of TCRαchains (Figure S2), the surface expression of TCRβ (Figure 3C) and CD45 (Figure 3D) [32] on DP thymocytes were observed between the genotypes. In addition, the expression levels of CD5, which is known to be correlated with the avidity of TCR/pMHC interaction [33], on DP, CD4SP and CD8SP cells were comparable between the genotypes (Figure 3E). These results strongly indicated that the αβTCR recombination and TCR/pMHC avidity are normal during T cell development in *Zfat* ^f/f^-*LckCre* mice.

To further confirm the impaired positive selection in *Zfat* ^f/f^-*LckCre* mice, we crossed ovalbumin (OVA)-specific MHC class I-restricted TCR transgenic (OT-I) [34] or MHC class II-restricted TCR transgenic mice (OT-II) [35] with *Zfat* ^f/f^-*LckCre* mice, and examined the developmental fate of thymocytes in OT-I *Zfat* ^f/f^-*LckCre* and OT-II *Zfat* ^f/f^-*LckCre* mice. It is known that DP cells pass through the CD4^+^CD8^int^ transitional stage before complete differentiation into either CD4SP or CD8SP cells [14]. As expected, not only the proportions but also the total numbers of CD8SP and CD4^+^CD8^int^ cells were significantly decreased in OT-I *Zfat* ^f/f^-*LckCre* mice compared with those of OT-I *Zfat* ^f/f^ mice (Figure 3F), suggesting that MHC class I-restricted positive selection in OT-I *Zfat* ^f/f^-*LckCre* mice was impaired. In addtion, MHC class II-restricted positive selection in OT-II *Zfat* ^f/f^-*LckCre* mice was also impaired, as both the proportions and the total numbers of CD4SP and CD4^+^CD8^int^ cells were remarkably reduced in OT-II *Zfat* ^f/f^-*LckCre* compared with those of OT-II *Zfat* ^f/f^ mice (Figure 3G). Together, these results confirmed that positive selection is impaired in the *Zfat*-deficient thymocytes.

### *Zfat*-deficiency Impaired CD3ζ Phosphorylation with Defects in ERK1/2 Activation

**Figure 4:**
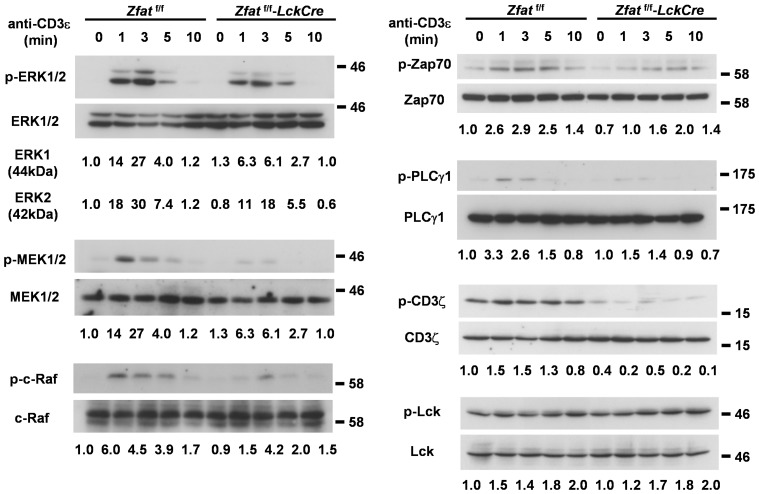
*Zfat-*deficiency impaired CD3ζ phosphorylation with defects in ERK1/2 activation. Immunoblots for phosphorylated or total protein of ERK, MEK1/2, c-Raf, Zap70, PLCγ1, CD3ζ and Lck before or at the indicated time points after the stimulation with cross-linking anti-CD3ε antibody in thymocytes from the indicated genotypes. The values below each image represent the relative ratio of the amount of phosphorylated protein to total protein. Data are representative of three independent experiments.

To elucidate mechanisms of the impaired positive selection observed in *Zfat* ^f/f^-*LckCre* DP cells, we examined phosphorylation of molecules working at the signaling transduced by TCR-stimulation. Phosphorylations of ERK1/2 induced by TCR-stimulation, which is known to be critical in the positive selection, were markedly decreased in *Zfat* ^f/f^-*LckCre* thymocytes compared with those of *Zfat* ^f/f^ thymocytes (Figure 4). In agreement with the defects in ERK1/2 activation, the phosphorylations of both MEK1/2 and c-Raf, which are located upstream of the ERK signaling pathway, were also reduced in the *Zfat* ^f/f^-*LckCre* thymocytes compared with those of *Zfat* ^f/f^ thymocytes (Figure 4). Furthermore, the phosphorylations of Zap70 and PLCγ1 were diminished in *Zfat* ^f/f^-*LckCre* thymocytes (Figure 4). Finally, TCR stimulation-induced phosphorylation of CD3ζ was virtually ablated in the *Zfat* ^f/f^-*LckCre* thymocytes, and the phosphorylated-CD3ζ at non-stimulated status was also apparently diminished due to the *Zfat*-deficiency (Figure 4). However, phosphorylated status at Tyr505 of Lck, which phosphorylates tyrosine residues in ITAMs of CD3ζ in response to TCR-stimulation [36], was comparable between the genotypes. Taken together, these results indicate TCR signaling cascade is strikingly attenuated in *Zfat* ^f/f^-*LckCre* DP cells at CD3ζ phosphorylation concurrent with ERK1/2 phosphorylation. Moreover, the impaired positive selection in *Zfat* ^f/f^-*LckCre* DP cells is thought to be attributed to defects in the activation of ERK1/2 induced by TCR-stimulation.

### TCR-stimulation-induced Egr Expressions were Impaired in the *Zfat*-deficient Thymocytes

**Figure 5:**
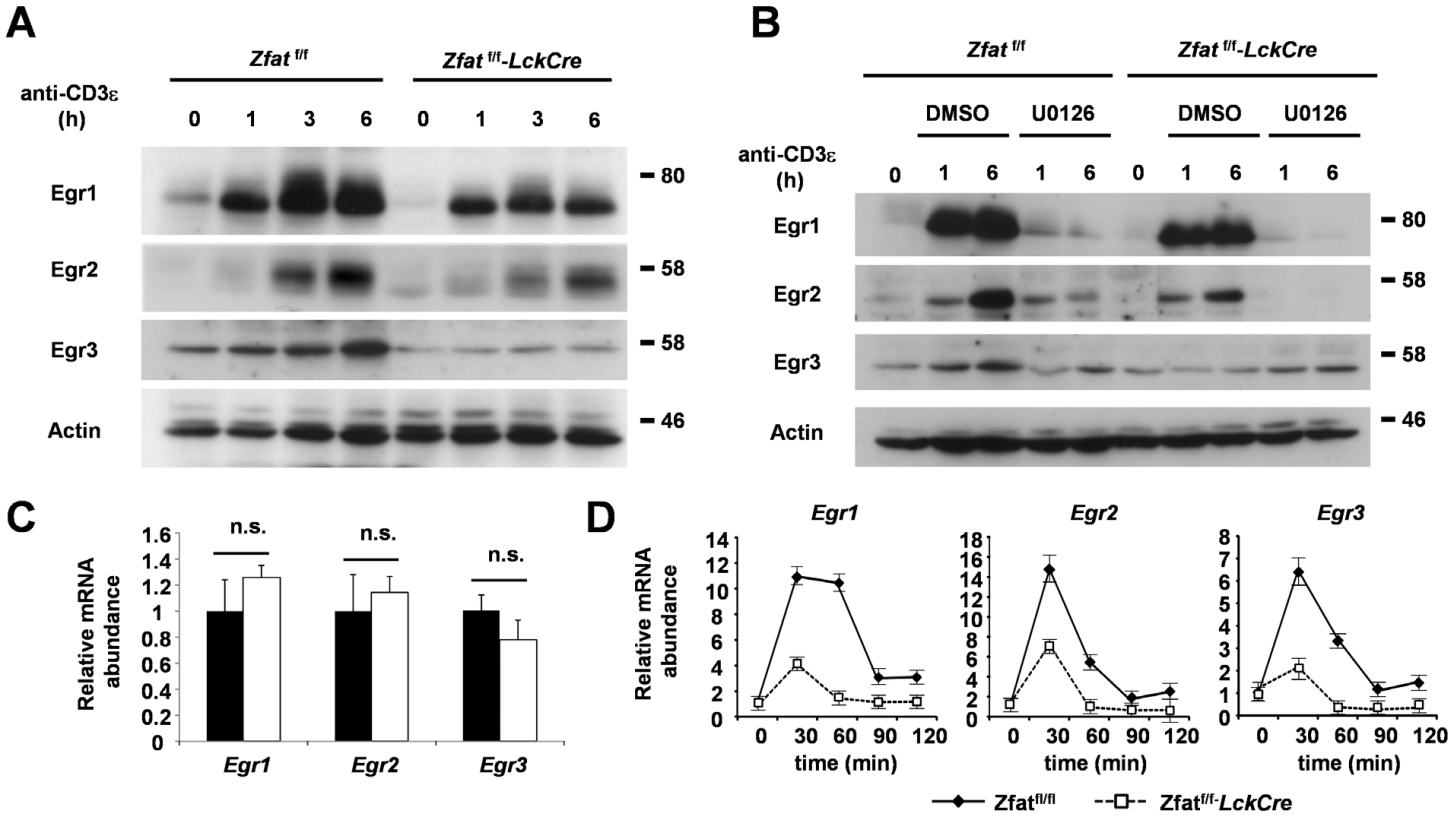
TCR-stimulation induced Egr expressions were impaired in the *Zfat*-deficient DP thymocytes. (A)Immunoblots for Egr1, Egr2 and Egr3 before or at the indicated time points after the stimulation with plate-bound anti-CD3ε and anti-CD28 antibodies in DP cells from the indicated genotypes. Actin was used as a loading control. Data are representative of three independent experiments. (B) Immunoblots for Egr1, Egr2 and Egr3 in DP cells from the indicated genotypes before or at the indicated time points after the stimulation with plate-bound anti-CD3ε and anti-CD28 antibodies under the condition with U0126 in DMSO or with DMSO alone. Actin was used as a loading control. Data are representative of three independent experiments. (C, D) Quantitative RT-PCR analysis of *Egr1*, *Egr2* and *Egr3* expression before (C) or at the indicated time points after the stimulation with anti-CD3ε and anti-CD28 antibodies (D) in DP cells from *Zfat* ^f/f^ and *Zfat* ^f/f^-*LckCre* mice. The relative expression for each gene was normalized by the expression of *Actb*. The results are presented as the value relative to the unstimulated DP cells from *Zfat* ^f/f^ mice. The data are the mean ± s.d.; n.s., not significant.

Next, we examined the expression levels of Egr1, Egr2 and Egr3, which are known to be induced by TCR-stimulation. Both the Egr1 and Egr2 expressions were robustly increased in the *Zfat* ^f/f^ DP cells after the stimulation with anti-CD3ε and anti-CD28 antibodies *in vitro* (Figure 5A), whereas expression levels of both Egr1 and Egr2 in the *Zfat* ^f/f^-*LckCre* DP cells after the stimulation were not much induced compared with those of *Zfat* ^f/f^ DP cells. In addition, it appeared that the Egr1 expression level before the stimulation was also decreased in the *Zfat* ^f/f^-*LckCre* DP cells compared with that of the *Zfat* ^f/f^ DP cells (Figure 5A). On the other hand, Egr3 was constitutively expressed and slightly increased in the *Zfat* ^f/f^ DP cells after the stimulation, whereas the expression levels of Egr3 in the *Zfat* ^f/f^-*LckCre* DP cells were constitutively lower compared with those of *Zfat* ^f/f^ DP cells and rarely enhanced by TCR-stimulation (Figure 5A). These results indicated that *Zfat*-deficiency causes dysregulated expression of Egr protein in the DP cells before and after TCR-stimulation. Similar experiments were performed on the thymocytes from OT-II *Zfat* ^f/f^-*LckCre* mice. Phosphorylation of ERK induced by TCR-stimulation in the thymocytes from OT-II *Zfat* ^f/f^-*LckCre* mice was decreased compared with that of OT-II *Zfat* ^f/f^ mice (Figure S3A). Furthermore, the levels of TCR-stimulation-induced Egr1, Egr2 and Egr3 expression in OT-II *Zfat* ^f/f^-*LckCre* thymocytes were all decreased compared with those of OT-II *Zfat* ^f/f^ mice (Figure S3B), and together these findings suggested that the impaired phosphorylation of ERK and Egr expression induced by TCR-stimulation was caused by molecules other than TCR itself.

To address whether MEK activation is essential for ERK mediated Egr inductions under the TCR-stimulated condition, we stimulated DP cells in the presence of U0126, an inhibitor of MEK1/2 [37]. We found that the inductions of Egr1 and Egr2 were considerably reduced by the treatment with U0126 both in *Zfat* ^f/f^ and *Zfat* ^f/f^-*LckCre* DP cells (Figure 5B), indicating that the enhancements of Egr1 and Egr2 expression by TCR-stimulation were essentially regulated by the MEK-ERK axis. On the other hand, the Egr3 induction was just partly reduced by the treatment with U0126 in *Zfat* ^f/f^ DP cells, whereas the Egr3 expression in the *Zfat* ^f/f^-*LckCre* DP cells seemed to be slightly increased or unaltered by TCR-stimulation in the presence of U0126 (Figure 5B). These results suggested the possibility that Egr3 expression may be regulated by other signaling pathways in addition to the MEK-ERK pathway.

While the levels of *Egr* mRNA expression in the unstimulated DP cells were comparable between the *Zfat* ^f/f^ and *Zfat* ^f/f^-*LckCre* genotypes (Figure 5C), each of the *Egr1*, *Egr2* and *Egr3* mRNA inductions by TCR-stimulation were robustly suppressed in *Zfat* ^f/f^-*LckCre* DP cells compared with those of *Zfat* ^f/f^ DP cells (Figure 5D), suggesting that Zfat is required for proper *Egr* gene expression induced by TCR-stimulation. However, considering the fact that the expressions of Egr proteins were slightly decreased in the unstimulated *Zfat* ^f/f^-*LckCre* DP cells (Figure 5A), *Zfat*-deficiency might affect the turnover or degradation of Egr proteins as well as the expression of *Egr* mRNAs. Taken together, these results indicated that impairment of the TCR-stimulation-induced Egr inductions in the *Zfat*-deficient thymocytes leads to the defects of positive selection.

## Discussion

In this study, we demonstrated that Zfat is required for the proper phosphorylation of CD3ζ, and is a critical regulator for T cell development in the thymus, especially for positive selection. The molecular mechanism by which *Zfat*-deficiency leads to impaired positive selection would be mainly dependent on the loss of CD3ζphosphorylation leading to the impaired ERK activation transduced by TCR-stimulation. Furthermore, activated ERK-mediated expression of Egr1 and Egr2, which are critical regulators for positive selection, were also decreased in the *Zfat* ^f/f^-*LckCre* thymocytes. Taken together, these results suggest that Zfat is involved in the proper regulation of the TCR-proximal signalings, which is required for positive selection in the thymus.

*Zfat* ^f/f^-*LckCre* mice showed a considerable reduction in the number of DP thymocytes. We could not find apparent defects in T cell development at DN stage or transition of DN to DP cells in *Zfat*-deficient thymocytes *in vivo* and *ex vivo* experiments. Therefore, the reduction in the number of DP cells in *Zfat* ^f/f^-*LckCre* mice may be due to the *Zfat*-deficiency at the DP stage. However, Zfat expression was not completely abolished in the *Zfat* ^f/f^-*LckCre* DN3 thymocytes, and thus the possibility that Zfat is necessary for proper development at the DN stage cannot be excluded.

Impaired positive selection in *Zfat*-deficient thymocytes was not restored in the presence of OT-I TCR or OT-II TCR transgene, which promote positive selection and differentiation of DP cells into CD8SP or CD4SP cells. These results strongly indicated that positive selection is impaired in *Zfat*-deficient mice, and also suggested that the defect of positive selection is caused by the dysregulated signaling downstream of TCR itself. TCR-stimulation triggers activation of the ERK pathway through sequential activation of Ras, Raf and MEK [17]. Activation of ERK is critical in the TCR-mediated signaling cascades and an essential requirement for positive selection in the thymus [19], [20].

Both Egr1 and Egr2 expression are critically regulated by activated ERK transduced by TCR-stimulation and play pivotal roles in positive selection and survival of DP cells [24], [25], [38]. Intriguingly, we identified the defects of TCR-stimulated ERK phosphorylation and Egr inductions in *Zfat*-deficient DP thymocytes, indicating that impaired Egr induction was at least partially responsible for the defects of positive selection in *Zfat* ^f/f^-*LckCre* mice. Moreover, Egr3 was also dysregulated in *Zfat*-deficient thymocytes. *Egr3*-deficient mice have been reported to exhibit a defect in the thymocytes proliferation and a partial block in differentiation at the DN3 stage [26]. Therefore, a possibility that Zfat plays an important role in the proliferation of thymocytes during the DN to DP transition through Egr3 induction is not excluded.

Decreased phosphorylation of CD3ζ in *Zfat* ^f/f^-*LckCre* thymocytes induced by TCR-stimulation was observed, indicating that *Zfat*-deficiency results in impaired activation of TCR signaling at proximal level. Tyrosines in ITAMs of CD3ζ are phosphorylated by Src family kinase Lck, and then the tyrosine-phosphorylated ITAMs of CD3ζ serve as docking sites for Zap70 in response to TCR stimulation [36]. However, *Zfat*-deficiency did not affect phosphorylation status of Lck in the thymocytes, whereas phosphorylation of Zap70 was reduced in *Zfat* ^f/f^-*LckCre* thymocytes. We have not elucidated how exactly Zfat affects the CD3ζphosphorylation in this study. Activation of CD3ζ is negatively regulated by SHP−1 and SHP−2 (SH2 domain-containing phosphatase-1 and -2) through dephosphorylation [39], [40]. Moreover, c-Cbl E3 ubiquitin ligase reduces CD3ζ levels at the plasma membrane by stimulating internalization [39], [41]. Considering that Zfat is expected to be a transcriptional regulator in the nucleus [2], [3], Zfat might affect the expressions of the genes involved in the regulation of CD3ζ phosphorylation, such as SHP-1, SHP-2 and c-Cbl. However, elucidation of the precise molecular mechanisms of Zfat function in regulation of TCR signaling should await future studies.

Proper activation of TCR signaling is also required for negative selection in the thymus. Thus, Zfat might be involved in the negative selection since *Zfat*-deficiency results in the defect in proximal molecules of TCR complex. However, we have not seen obvious differences in the T cell developments between H-Y *Zfat* ^f/f^ *and* H-Y *Zfat* ^f/f^-*LckCre* male mice in preliminarily experiments (data not shown). To establish a role for Zfat in the negative selection, further studies should be required in the future.

In conclusion, we demonstrated that *Zfat*-deficiency in DP cells results in a loss of CD3ζ phosphorylation with dysregulation of ERK and Egr activities leading to impaired positive selection in the thymus, suggesting that Zfat is a pivotal molecule in T cell development. As the activation of ERK-Egr pathway was not completely impaired in the *Zfat*-deficient thymocytes, the possibility that Zfat plays crucial roles in particular signaling pathways other than ERK-mediated pathway does exist. Thus, a full understanding of the roles and precise molecular mechanisms of the transcriptional regulator Zfat will lead to a better understanding of the orchestrated gene expression programs and provide deeper insight into T cell development, immune regulation and a wide variety of diseases.

## Materials and Methods

### Mice

*Zfat* ^f/f^ mice were originally generated as described previously [6] and backcrossed to a C57BL/6 background over seven generations. *Zfat* ^f/f^ mice were crossed to Lck-Cre mice from Taconic to generate T-cell-specific *Zfat* knockout (*Zfat* ^f/f^-*LckCre*) mice in the C57BL/6 background. All the animal experiments were approved by the Animal Care and Use Committee of the NCGM Research Institute, and the experiments on mice were carried out under the guidelines of the Institutional Animal Care and Use Committee of Fukuoka University.

### Immunoblotting

The nuclear and cytoplasmic fractions from thymocytes were prepared using NE-PER Nuclear and Cytoplasmic Extraction Kit (Pierce). Separated cells were lysed in RIPA buffer [50 mM Tris-HCl, pH 7.5, 150 mM NaCl, 1% NP−40, 0.5% deoxycholate, 0.1% SDS, protease inhibitor cocktail (Roche)]. Equal amounts of total lysates were electrophoresed in 7, 8.5 or 4–20% SDS-polyacrylamide gels, and transferred to a nitrocellulose membrane (GE Healthcare). The antibodies used for immunoblotting analysis and their specificities were as follows: Egr3, phosphorylated ERK, antibodies for phosphorylated and total Zap70, PLCγ1, c-Raf, and Lck (all from Cell Signaling Technology); CREB1 (C-21), Erk (K-23) and Egr1 (C-19; all from Santa Cruz); total and phosphorylated MEK1/2 (from New England Biolabs); Actin (A2066; from Sigma); Egr2 (from Proteintech Group); CD3ζ (from Exbio); phosphorylated CD3ζ (A0468; from Assay biotech). Anti-Zfat antibody was prepared as described previously [2]. The horseradish peroxidase-conjugated secondary antibody (GE Healthcare) and SuperSignal West Pico Chemiluminescent Substrate (Thermo Scientific) were used for the detection. The quantitative analysis of the immunoblotting was done using the integration value of each blot with Image J software (version1.46, US National Institutes of Health) [42].

### Flow Cytometry

Cells from the spleen, lymph nodes and the thymus were depleted of erythrocytes by hypotonic lysis and stained with fluorophore-conjugated antibodies. A Cytofix/Cytoperm kit (BD Biosciences) was used for analysis of the intracellular detection of TCRβ. Data were collected with a cytometer (FACSAria II; BD Biosciences) and analyzed with FlowJo software (Tomy Digital Biology). The fluorophore-conjugated antibodies used for flow cytometry analysis and their specificities were as follows: CD4 (RM4–5), CD8 (53-6.7), B220 (RA3-6B2), CD25 (PC61), CD44 (IM7), CD27 (LG.3A10), TCRβ (H57-597), CD69 (H1.2F3), CD45 (30-F11) and CD5 (53-7.3; all from Biolegend).

### *In vitro* Differentiation of DN3 Cells

Tst-4/DLL1 stromal cells (RCB2118) were provided by the RIKEN BRC through the National Bio-Resource Project of the MEXT, Japan. Cells were plated and co-cultured with sorted DN4 (CD4^−^CD8^−^CD25^−^CD44^−^) cells. DN4 cells (1×10^5^) were cultured at 37°C with 5% CO_2_ in RPMI 1640 medium containing 10% FCS, penicillin-streptomycin-glutamine (Life Technologies) and β-mercaptoethanol (50 nM; Sigma) supplemented with recombinant mouse IL-7 (0.5 ng/ml; Pepro Tech) and SCF (1 µg/ml; R&D Systems).

### *In vitro* TCR-stimulation

Thymocytes (1×10^7^) from mice were stimulated at 37°C with anti-CD3ε antibody (10 µg/ml, 145-2C11; Biolegend) cross-linked with goat anti-hamster IgG (80 µg/ml; Southern Biotech) in RPMI 1640 medium containing 10% FCS, penicillin-streptomycin-glutamine (Life Technologies) and β-mercaptoethanol (50 nM; Sigma). For the stimulation with plate-bound anti-CD3ε and anti-CD28 antibodies, plates were coated overnight with anti-CD3ε (1 µg/ml) and anti-CD28 (3 µg/ml, 37.51; Biolegend) antibodies. DP cells (5×10^6^) were stimulated with plate-bound anti-CD3ε and anti-CD28 antibodies under the condition with U0126 (100 µM; Calbiochem) in DMSO or with DMSO alone.

### Quantitative RT-PCR

Total RNA was extracted by TRIZol reagent with PureLink RNA Mini Kit (Life Technologies). Superscript VILO cDNA synthesis kit (Life Technologies) was used for reverse transcription. Quantitative RT-PCR was performed by using Thunderbird SYBR qPCR Mix (TOYOBO) with ABI PRISM 7900HT (Applied Biosystems). The PCR primers used for *Egr1* 5′-AGGACTTGATTTGCATGGTATTGGA-3′ and 5′-ATGCAGGGCAGGGTTCTGAG-3′, *Egr2* 5′-GAACCAGGACACCGTGAGATGA-3′ *and* 5′-GTAGTGTTGGCAGCTCGGACAG-3′, *Egr3* 5′-GACTCGGTAGCCCATTACAATCAGA-3′ and 5′-GAGAGTTCCGGATTGGGCTTC-3′ and *Actin* 5′-CATCCGTAAAGACCTCTATGCCAAC-3′ and 5′-ATGGAGCCACCGATCCACA-3′.

### Statistical Analysis

Data are presented as the means ± s.d. and statistical analysis was performed using an unpaired Student’s *t*-test when comparing the means of two groups. Differences of *P*<0.05 were considered to be statistically significant (*P<0.05; **P<0.01).

### Accession Number

GenBank mRNA sequences: Zfat, NT_078782.

## Supporting Information

Figure S1 **Surface expression of IL-7Rα and intracellular expression of Bcl-2.** Flow cytometry analysis of the expression of surface IL-7Rα (A) and intracellular Bcl-2 (B) in the DP, CD4SP and CD8SP cells from *Zfat* ^f/f^ (gray-filled) and *Zfat* ^f/f^-*LckCre* (black line) mice at 6 to 7 weeks of age (left). Data of thymocytes from *Zfat* ^f/f^ (black bar) and *Zfat* ^f/f^-*LckCre* (white bar) mice were measured as the mean fluorescence intensity (MFI) (right). The fluorophore-conjugated antibodies used for flow cytometry analysis and their specificities were as follows: IL-7Rα (A7R34) and Bcl-2 (10C4; all from Biolegend). Data are representative of three independent experiments. The data are the mean ± s.d.; * P<0.05; ** P<0.01; n.s., not significant.
(TIF)

Figure S2 **Rearrangements of TCRα chains in** ***Zfat*** **^f/f^ or** ***Zfat*** **^f/f^-** ***LckCre*** **thymocytes.** Semiquantitative RT-PCR analysis of *Vα*-to-*Cα* rearrangements in DP thymocytes from the indicated genotypes. *Cα-Cα* amplification within a *Cα* region served as the control. *Gapdh*, an internal control. Primer sequences used for amplifications were as follows: *Vα3* 5′-CCCAGTGGTTCAAGGAGTGA-3′, *Vα6* 5′-CTGACTCATGTCAGCCTGAGAG-3′, *Vα8* 5′-CAACAAGAGGACCGAGCACC-3′, *Vα14* 5′-TGGGAGATACTCAGCAACTCTGG-3′, *Vα19* 5′-CTGCTTCTGACAGAGCTCCAG-3′ and *Cα* 5′-TTCAAAGAGACCAACGCCAC-3′ with *Cα* Rv primer 5′-TTCAGCAGGAGGATTCGGAG-3′, *Gapdh* Fw 5′-GAACGGATTTGGCCGTATTG-3′ and *Gapdh* Fw 5′-GATGATGACCCTTTTGGCTC-3′. Data are representative of three independent experiments.
(TIF)

Figure S3 **Reduced ERK activation and Egr induction in OT-II** ***Zfat*** **^f/f^** ***-LckCre*** **thymocytes.** (A) Immunoblots for phosphorylated or total protein of ERK before or the indicated time points after the stimulation with cross-linking anti-CD3ε antibody in thymocytes from the indicated genotypes. The values below each image represent the relative ratio of the amount of phosphorylated protein to total protein. Data are representative of three independent experiments. (B) Immunoblots for Egr1, Egr2 and Egr3 before or at the indicated time points after the stimulation with plate-bound anti-CD3ε and anti-CD28 antibodies in DP cells from the indicated genotypes. Actin was used as a loading control. Data are representative of three independent experiments.
(TIF)
