# Supplementary figures and images for: Zfat-Deficiency Results in a Loss of CD3ζ Phosphorylation with Dysregulation of ERK and Egr Activities Leading to Impaired Positive Selection
Source: PLoS One. 2013 Oct 3;8(10):e76254. doi: 10.1371/journal.pone.0076254 (PMC3789737; doi:10.1371/journal.pone.0076254)

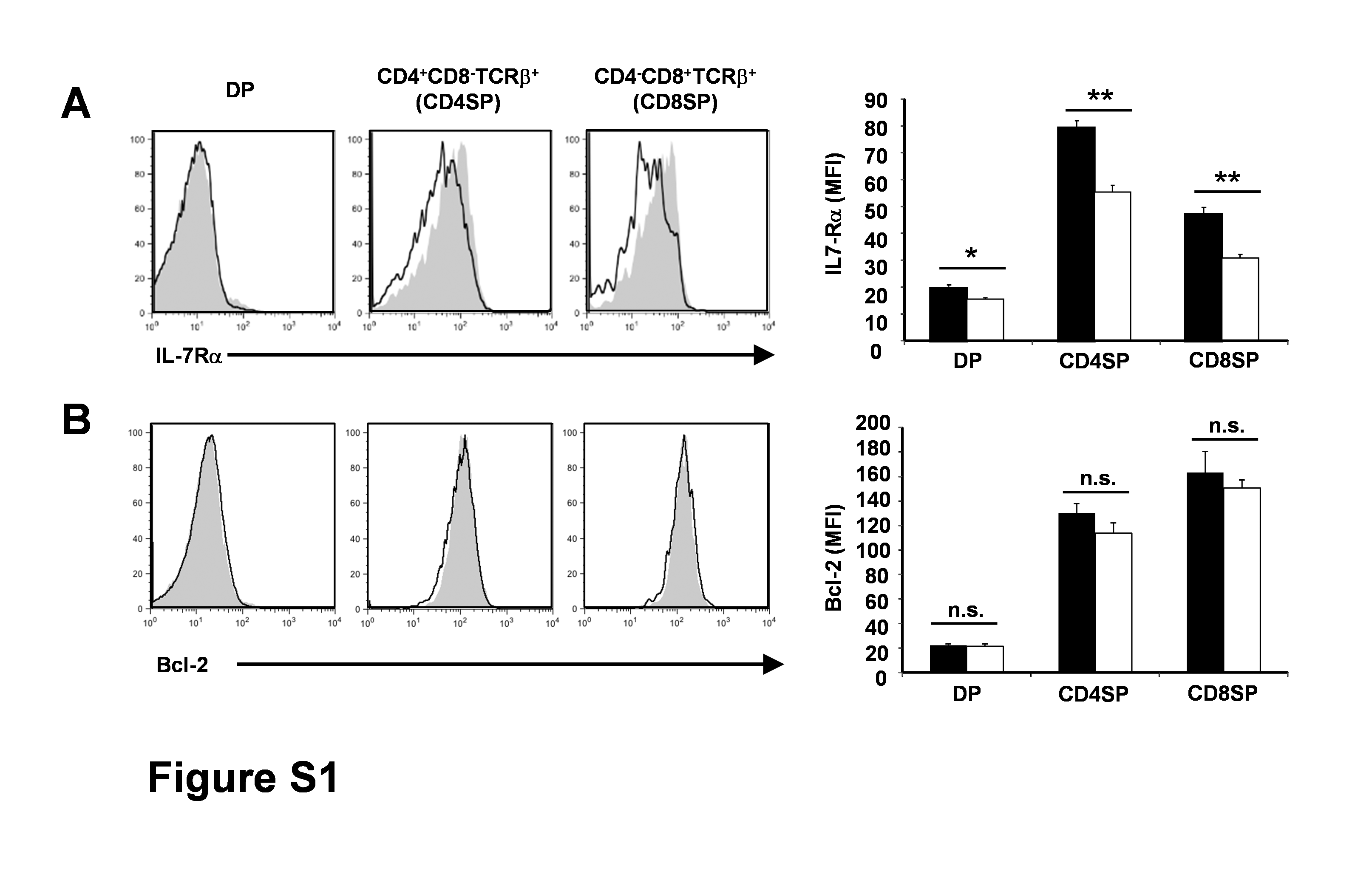

Supplement: Figure S1 — Surface expression of IL-7Rα and intracellular expression of Bcl-2. Flow cytometry analysis of the expression of surface IL-7Rα (A) and intracellular Bcl-2 (B) in the DP, CD4SP and CD8SP cells from Zfat f/f (gray-filled) and Zfat f/f-LckCre (black line) mice at 6 to 7 weeks of age (left). Data of thymocytes from Zfat f/f (black bar) and Zfat f/f-LckCre (white bar) mice were measured as the mean fluorescence intensity (MFI) (right). The fluorophore-conjugated antibodies used for flow cytometry analysis and their specificities were as follows: IL-7Rα (A7R34) and Bcl-2 (10C4; all from Biolegend). Data are representative of three independent experiments. The data are the mean ± s.d.; * P<0.05; ** P<0.01; n.s., not significant. (TIF) [file pone.0076254.s001.tif]

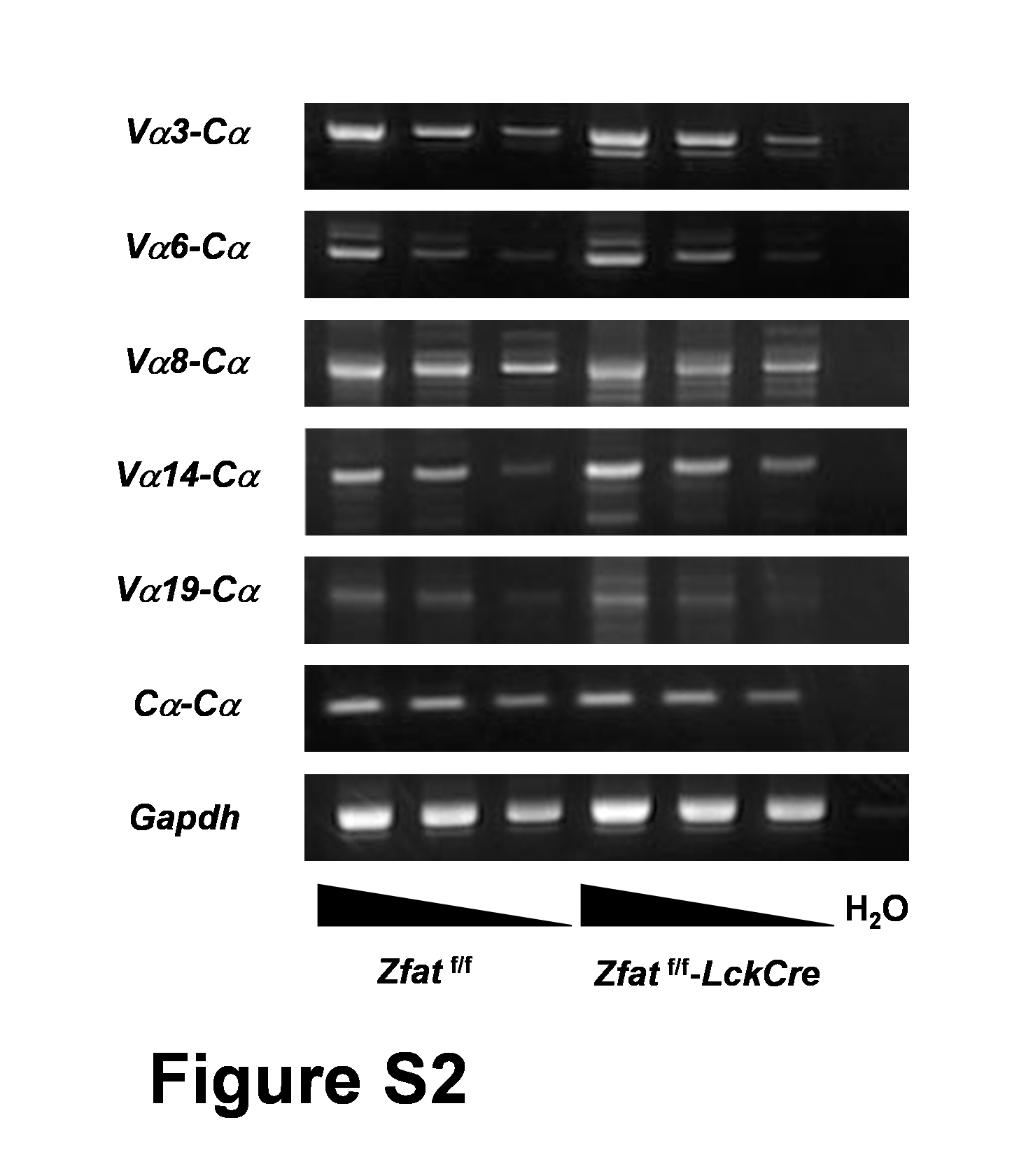

Supplement: Figure S2 — Rearrangements of TCRα chains in Zfat f/f or Zfat f/f- LckCre thymocytes. Semiquantitative RT-PCR analysis of Vα-to-Cα rearrangements in DP thymocytes from the indicated genotypes. Cα-Cα amplification within a Cα region served as the control. Gapdh, an internal control. Primer sequences used for amplifications were as follows: Vα3 5′-CCCAGTGGTTCAAGGAGTGA-3′, Vα6 5′-CTGACTCATGTCAGCCTGAGAG-3′, Vα8 5′-CAACAAGAGGACCGAGCACC-3′, Vα14 5′-TGGGAGATACTCAGCAACTCTGG-3′, Vα19 5′-CTGCTTCTGACAGAGCTCCAG-3′ and Cα 5′-TTCAAAGAGACCAACGCCAC-3′ with Cα Rv primer 5′-TTCAGCAGGAGGATTCGGAG-3′, Gapdh Fw 5′-GAACGGATTTGGCCGTATTG-3′ and Gapdh Fw 5′-GATGATGACCCTTTTGGCTC-3′. Data are representative of three independent experiments. (TIF) [file pone.0076254.s002.tif]

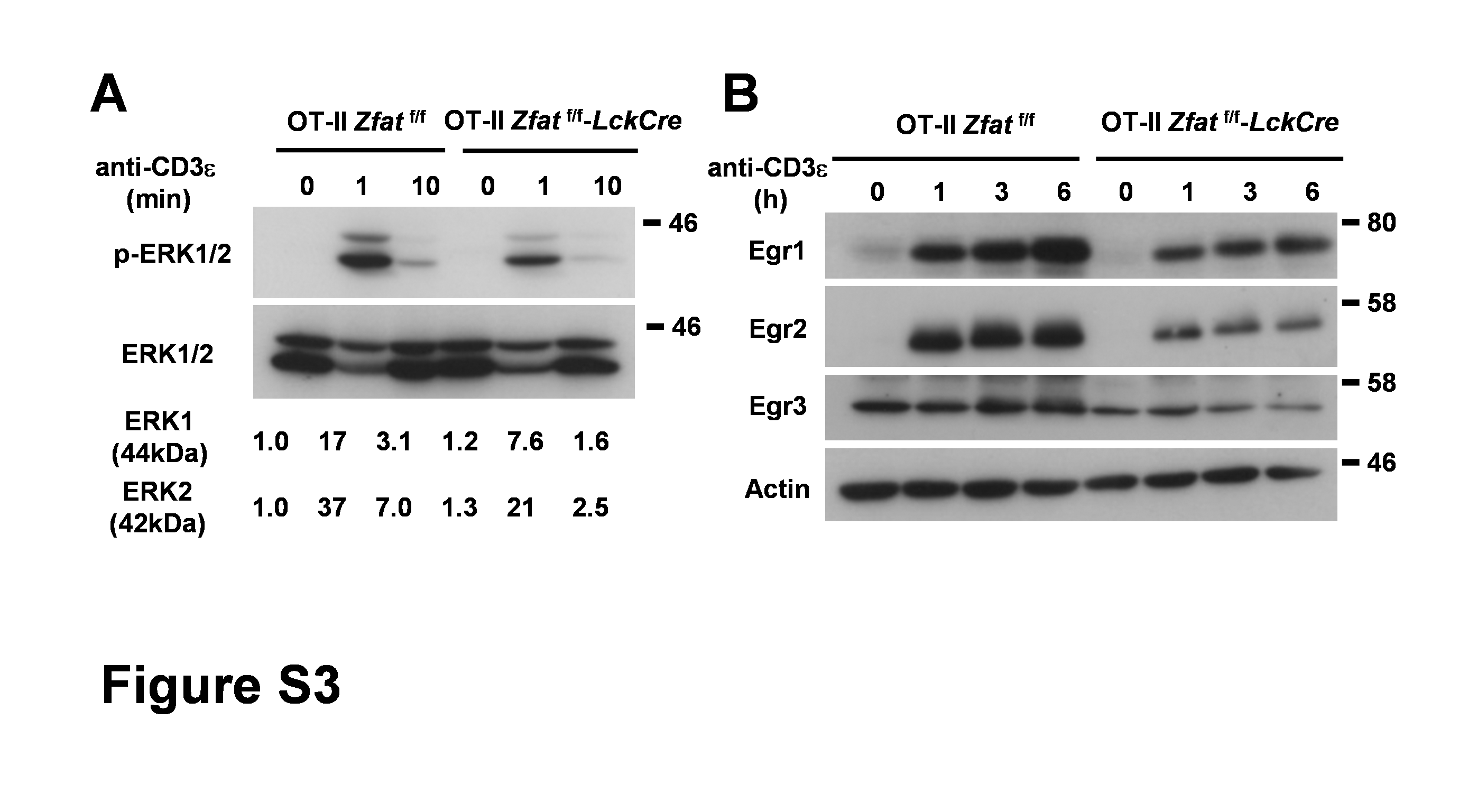

Supplement: Figure S3 — Reduced ERK activation and Egr induction in OT-II Zfat f/f -LckCre thymocytes. (A) Immunoblots for phosphorylated or total protein of ERK before or the indicated time points after the stimulation with cross-linking anti-CD3ε antibody in thymocytes from the indicated genotypes. The values below each image represent the relative ratio of the amount of phosphorylated protein to total protein. Data are representative of three independent experiments. (B) Immunoblots for Egr1, Egr2 and Egr3 before or at the indicated time points after the stimulation with plate-bound anti-CD3ε and anti-CD28 antibodies in DP cells from the indicated genotypes. Actin was used as a loading control. Data are representative of three independent experiments. (TIF) [file pone.0076254.s003.tif]
